# Activation of bacterial channel MscL in mechanically stimulated droplet interface bilayers

**DOI:** 10.1038/srep13726

**Published:** 2015-09-08

**Authors:** Joseph S. Najem, Myles D. Dunlap, Ian D. Rowe, Eric C. Freeman, John W. Grant, Sergei Sukharev, Donald J. Leo

**Affiliations:** 1 Department of Mechanical Engineering, Virginia Polytechnic Institute and State University, Blacksburg, Virginia 24061, United States.; 2 School of Biomedical Engineering and Sciences, Virginia Polytechnic Institute and State University, Blacksburg, Virginia 24061, United States; 3 Department of Engineering Science and Mechanics, Virginia Polytechnic Institute and State University, Blacksburg, Virginia 24061, United States; 4 Department of Biology, University of Maryland, College Park, Maryland 20742, United States; 5 College of Engineering, University of Georgia, Athens, Georgia 30602, United States

## Abstract

MscL, a stretch-activated channel, saves bacteria experiencing hypo-osmotic shocks from lysis. Its high conductance and controllable activation makes it a strong candidate to serve as a transducer in stimuli-responsive biomolecular materials. Droplet interface bilayers (DIBs), flexible insulating scaffolds for such materials, can be used as a new platform for incorporation and activation of MscL. Here, we report the first reconstitution and activation of the low-threshold V23T mutant of MscL in a DIB as a response to axial compressions of the droplets. Gating occurs near maximum compression of both droplets where tension in the membrane is maximal. The observed 0.1–3 nS conductance levels correspond to the V23T-MscL sub-conductive and fully open states recorded in native bacterial membranes or liposomes. Geometrical analysis of droplets during compression indicates that both contact angle and total area of the water-oil interfaces contribute to the generation of tension in the bilayer. The measured expansion of the interfaces by 2.5% is predicted to generate a 4–6 mN/m tension in the bilayer, just sufficient for gating. This work clarifies the principles of interconversion between bulk and surface forces in the DIB, facilitates the measurements of fundamental membrane properties, and improves our understanding of MscL response to membrane tension.

MscL channels, intrinsic components of the cytoplasmic membrane in most bacteria, act as osmolyte release valves in response to increased membrane tension^1^,^2^. Multiple channels present in a small cell^3^ can generate a massive permeability response that saves the bacteria from lysis in the event of a hypo-osmotic shock^4^. MscL has been well characterized biophysically, mainly by use of the patch clamp technique^5^,^6^,^7^. The crystal structure of the MscL homolog^8^,^9^, combined with modelling^10^, and supported by different types of experimental data^11^,^12^,^13^,^14^, gave foundation for plausible structural models of its gating mechanism^13^,^15^. *E. coli* MscL is a homo-pentamer of two transmembrane domain subunits, each 136 amino acids long. The closed channel is a tight bundle of transmembrane helices, which under ~10 mN/m tension applied through the surrounding lipid bilayer, transforms into a ring of highly tilted helices forming a ~28 Å water-filled conductive pore^5^,^13^,^16^. The tight hydrophobic gate located at the intersection of the inner TM1 domains has been identified as the major determinant of the activation threshold^17^. Moreover, it has been shown that by making this constriction more hydrophilic, it is possible to lower the activation threshold^6^,^18^, allowing the engineering of more sensitive, or even spontaneously opened variants of the channel. Based on chemical or photochemical MscL activation, various designs of controllable valves were proposed^19^,^20^, primarily for the purpose of drug delivery. The combination of a relatively simple channel design and a large-scale conformational transition makes MscL appealing for bio-inspired stimuli-responsive material designs.

To date, the patch clamp technique, typically used in combination with a pressure clamp device delivering controllable suction to the pipette, has been the only approach to recording electrophysiological activities of MscL with high-resolution in both native bacterial membranes, as well as in reconstituted proteoliposomes^21^. In regard to using artificial systems suitable for engineering sensors, there have been major difficulties delivering sufficient tension to gate MscL. Planar lipid bilayers proved impractical due to the low inherent tensions in the range of 0.1–2 mN/m^22^ defined by the meniscus, also known as the Gibbs-Plateau region. Additionally, one attempt to reconstitute MscL in supported bilayers resorted to activation of channels solely by voltage^23^.

Droplet interface bilayers are flexible insulating scaffolds for stimuli-responsive molecular elements^24^ that can be used as a new platform for prospective studies of MscL activation. Compared to the patch clamp technique, the DIB system is miniature with the ability to control the composition of each membrane side. DIBs were first reported by Funakoshi *et al.*^25^, and later modified by Holden *et al.*^26^, as well as the Leo research group^27^. The DIB offered a wide range of functionalities that can be used to construct novel biomolecular systems appropriate for applications such as drug delivery^28^, high throughput screening^29^, and protein activity testing^26^. A recent attempt to reconstitute MscL in droplet interface bilayers utilized a cysteine mutant that was activated by charged sulfhydryl reagents without any mechanical input^30^.

In this article, we report the functional reconstitution of the V23T low-threshold MscL mutant in DIBs. We also report the first MscL activation in a DIB system, in response to an external mechanical stimulus transmitted to the bilayer through dynamic mechanical excitation. V23T-MscL, which was 6-His tagged to ease purification, activates at about half-tension (6-7 mN/m)^6^, when compared to WT-MscL (10–12 mN/m)^5^. An original test setup^31^ is designed to record V23T-MscL gating in response to micron-sized dynamic droplet distortions. This new technique of mechanical stimulation of the DIB opens more opportunities for studies of mechanoelectrical transduction in MscL channels. Our major result is reliable activation of MscL in DIBs subjected to periodic computer-controlled mechanical perturbation. To our surprise, activation is observed only in a narrow range of frequencies near 0.2 Hz, which prompted us to look at the factors that could define tension in the interfacial bilayer produced by the mechanical deformation of the system. We therefore discuss the basic principles of interconversion of bulk pressures into interfacial forces in DIB systems, including all its advantages and limitations. Our work has important implications for identifying the mechanoelectric transduction properties of the DIB, and on developing novel bio-inspired stimuli responsive materials^24^.

## Results

### Mechanically stimulated DIBs and low-threshold MscL mutant

**Figure 1:**
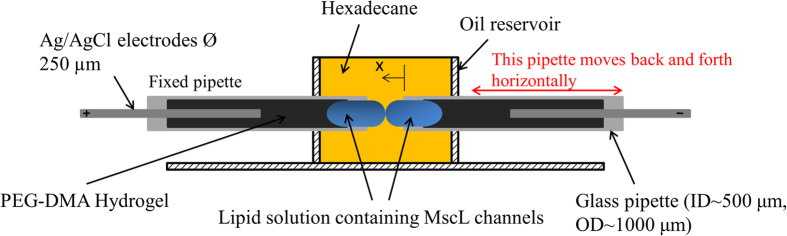
The experimental setup used to form the droplet interface bilayers, mechanically stimulate the droplets, and study MscL activity. The DIB supporting part consists of two hydrogel filled glass capillaries with inserted Ag/AgCl electrodes. Two lipid containing droplets are anchored to the tips of the micropipettes (ID ~500 μm, OD ~1000 μm), and placed within a cylindrical oil filled reservoir. The micropipette on the left is fixed and attached to the headstage of the Axopatch 200B amplifier, while the micropipette on the right is connected to a piezoelectric actuator which allows it to move horizontally.

**Figure 2:**
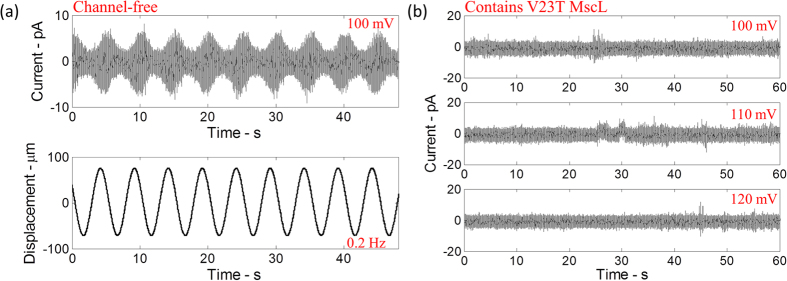
(**a**) The current response of the lipid bilayer (free of MscL channels) as droplets are oscillated at 0.2 Hz and 150 μm peak-to-peak amplitude. The current response is sinusoidal which corresponds to the change in the bilayer capacitance correlated with a change in the lipid bilayer area resulting from the sinusoidal oscillations of the droplets. (**b**) The current response of the lipid bilayer containing V23T-MscL channels when high transmembrane potentials are applied without mechanically stimulating the droplets.

A specialized test setup was developed to enable controllable actuation of the bilayer as well as accurate mechanical and electrical measurements of the interface. The test setup consisted of one droplet anchored to the tip of a mobile capillary mounted on a piezoelectric actuator and a second droplet anchored to a fixed substrate. The tension in the artificial lipid bilayer membrane is modulated by horizontally oscillating the droplet anchored to the piezoelectric actuator (Fig. 1), thereby distorting the shapes of the droplets (i.e. increasing the surface area of each droplet) and changing the contact angle between the water-oil interfaces. In this work, branched diphytanoyl phosphatidylcholine (DPhPC) is used to form bilayers. DPhPC bilayers neither oxidize nor exhibit phase transitions with temperature and thus are stable; they also have the advantage of high interfacial tension^32^. It is well established that the electrical properties of a lipid bilayer are modelled accurately by a high membrane resistance (typically in the giga-ohm range) in parallel with the membrane capacitance^27^. Therefore, low-frequency sinusoidal oscillations applied to the DIB result in a harmonic variation in bilayer capacitance that correlates with a change in the bilayer area (Fig. 2a). The electrical response of the DIB, free of MscL channels, was recorded simultaneously with video imaging of the droplets, while mechanically oscillated at frequencies ranging from 0.1 Hz up to 75 Hz, and peak-to-peak amplitudes ranging between 125 μm and 175 μm. These observations serve to form a basic understanding of the components of the mechanoelectrical response of the DIB without MscL, and serve to provide a ‘baseline’ control for the subsequent recordings with MscL channels reconstituted within the membrane. No measurable conductive component or gating-like spikes are observed in the control (Fig. 2a). This insures that the sub-conductive state events observed (when V23T-MscL is incorporated) are not simply random artefacts resulting from the bilayer oscillations and electrical recordings. The electrical responses of DIBs with V23T-MscL incorporated are also recorded in a broad range of transmembrane potentials (0–150 mV) with no mechanical stimulus applied (Fig. 2b). As previously observed, no gating-like spikes were recorded, indicating that creating tension in the membrane is essential for the gating of V23T-MscL. Note that all low amplitude (~5 pA) channel gating-like events, especially the ones in the 110 mV trace, likely represent transient conductive defects in the dynamic membrane structure stabilized by the electric field. With the droplets of approximately 0.5 mm in diameter, the initial bilayer area was approximately 0.0024 mm^2^. The generated currents in channel-free controls were small, reflecting a highly resistive lipid bilayer (~10 GΩ).

**Figure 3:**
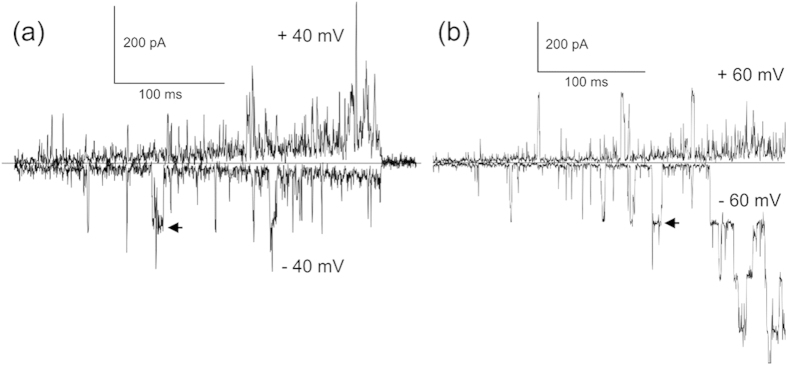
V23T-MscL activities recorded using standard patch-clamp technique in *E. coli* giant MJF465 spheroplasts (a) and in DPhPC liposomes reconstituted with purified protein (b). The mechanical stimuli in both cases are shallow ramps of negative pipette pressure (suction) to 50–170 mm Hg. Both traces recorded in a symmetric 400 mM KCl, 20 mM MgCl_2_ and 10 mM CaCl_2_ buffer characterized with the same conductivity as the buffer used for DIB formation. Conductance levels of 3.5–3.8 nS indicated by black arrows correspond to the fully open channels occurring amid various sub-conductive states.

In order to observe V23T-MscL-6His gating pattern in the native setting, we expressed the channel in MJF 465 *E. coli* cells^4^ and recorded traces from giant spheroplasts using standard patch-clamp technique. The gating response of the mutant in spheroplasts to mechanical stimuli (shallow negative pressure ramps ranging between 50 to 170 mm Hg) under an applied potential of  ±40 mV, are shown in Fig. 3a. In a parallel effort, we have purified 6-His tagged V23T-MscL and reconstituted it in DPhPC liposomes. Recordings from liposome ‘blisters’ were performed, with mechanical stimuli applied in a similar fashion as previously mentioned, in order to observe single-channel events (Fig. 3b). To compare conductance levels, both types of recordings were performed in the buffer of the same specific conductivity as used for DIBs. We found that the activities in both cases were essentially identical in terms of unitary conductance (3.5–3.8 nS full openings designated by arrows in Fig. 3), with similar kinetic patterns. When activated at relatively low open probability, V23T-MscL exhibits a variety of short-lived sub-conductive states. According to the previous data^6^,^33^, this mutant has a tension midpoint of 9.5 mN/m (compared to 12–14 mN/m for WT-MscL^33^), yet the first opening events are reproducibly observed near 6 mN/m. Based on this data, we anticipated that in DIBs V23T-MscL should start flickering at tensions between 6 and 7 mN/m.

### Transient MscL responses to harmonic compression

**Figure 4:**
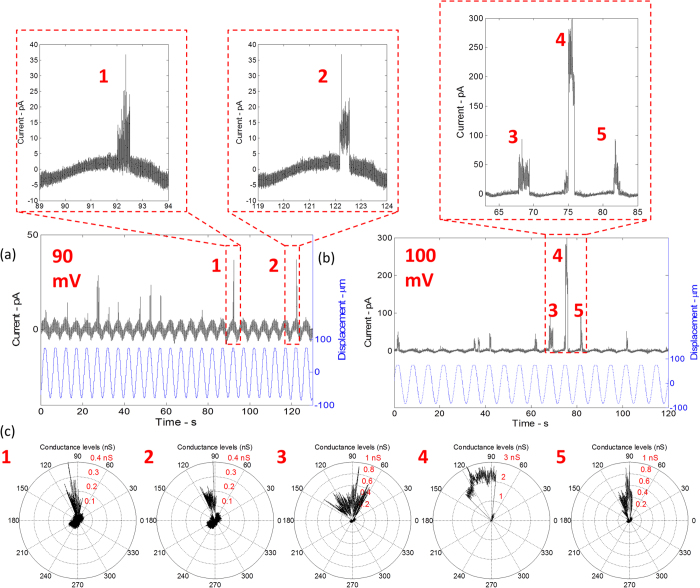
(**a,b**) The current response of the bilayer containing V23T-MscL mutant channels at 0.2 Hz (sinusoidal) and an applied transmembrane potential of 90 and 100 mV respectively. The shape of the current response is sinusoidal which corresponds to a sinusoidal change in bilayer capacitance as a consequence of the bilayer area change. The currents spikes at the peak of each cycle (i.e. maximum bilayer area) correspond to sub-conductance gating events of the MS channels. (**c**) Each of the six cycles is plotted in polar form indicating that the gating events consistently occur between 90 and 120 degrees.

The incorporation of V23T-MscL into DIBs is achieved by introducing proteoliposomes into both droplets. A low-threshold V23T mutant of MscL generates reliable activities including sub-conductive states as well as full opening events (Fig. 4a) when mechanically stimulated and a DC potential is applied to the membrane. These events are identical to those recorded using the patch-clamp technique from intact inner *E. coli* membranes and liposomes reconstituted with the purified V23T-MscL (Fig. 3). The mechanical stimulus is applied through axial sinusoidal displacements of one droplet toward and away from another with a frequency of 0.2 Hz and peak-to-peak amplitude of 150 μm. It is observed that the gating of V23T-MscL mostly occurs at transmembrane potentials above 80 mV and low oscillation frequencies (<1 Hz). Gating occurs exclusively near the point of shortest separation where both the contact area and fractional change in droplet area are maximal, while the lipid bilayer contact angle between the droplets (monolayers) is minimal.

To illustrate this observation, the representative DIB current traces, each covering one stimulation cycle, are plotted in the polar form indicating the position of the V23T-MscL opening events relative to the phase of the mechanical displacement (Fig. 4c). The results show that the channel consistently activates between 90 and 120 degrees. This region on the polar plot corresponds to the position near peak compression (where the sinusoidal mechanical input reaches maximum amplitude) and maximum bilayer area. The current is proportional to the capacitance of the bilayer 
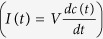
, which in turn obeys the same sine wave law (*C*(*t*)~*Asin*(*ωt*)) implying that there is a 90-degree phase shift between the actual change in bilayer area (i.e. capacitance) and the recorded capacitive current. This explains why the gating is seen in the 90–120 degree region on the current polar plot. Identical behaviour is observed when the potential is increased to 100 mV while a 0.2 Hz sinusoidal oscillation is maintained (Fig. 4b). The only difference seen is that the measured conductance levels are higher, which may indicate a greater expansion of the channel, due to an increased transmembrane potential.

**Figure 5:**
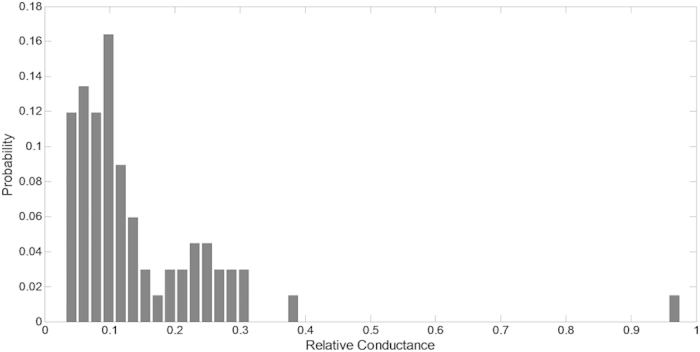
The amplitude histogram of V23T-MscL opening events obtained from multiple traces including 110 oscillation cycles. The droplets are subjected to periodic oscillation (0.2 Hz and 87.5 μm amplitude) and a transmembrane potential of 100 mV for several minutes and a total of 52 events were analysed. Under these conditions the probability of seeing an opening event per cycle was 0.47. The histogram shows that low-conductance sub-states are more likely to occur under the given testing conditions.

We have also analysed the amplitude distribution of opening events recorded over 110 cycles under ‘near-optimal’ stimulation with 0.2 Hz/87.5 μm sinusoidal droplet oscillations and a transmembrane potential of 100 mV (Fig. 5). Most of the events represent low sub-conductive states, consistent with low-open probability patch-clamp traces presented in Fig. 3 and previous analysis^6^. Full-openings of 3.1 nS amplitude constitute only 3% of all events.

### Effects of the displacement amplitude and applied transmembrane potential

**Figure 6:**
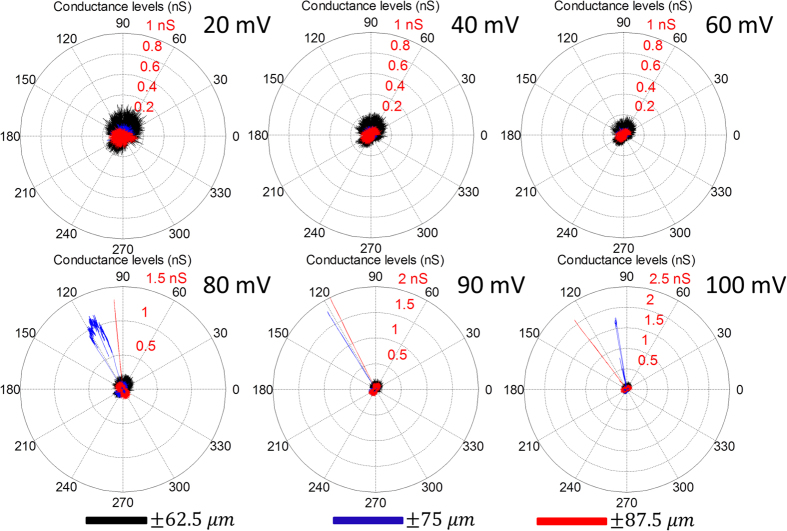
Gating dependence on the amplitude of droplet displacement and voltage. The droplets are oscillated at three different amplitudes. At each amplitude the applied electric potential is varied between 20 mV and 100 mV. The results in black, corresponding to the lowest amplitude (±65 μm), and show that no MS channel activity occur at all potentials. However, as the amplitude is increased to  ±75 μm, gating events occur at potentials starting from 80 mV up to 100 mV. The results at the highest amplitude (±87.5 μm) are similar to the previous case however the conductance levels are higher which may be a result of opening the MS channels further as the tension in the bilayer is higher at higher oscillation amplitudes.

The gating of the V23T-MscL channels is observed to be dependent on the transmembrane electrical potential, as well as the amplitude of oscillations. These findings are highlighted in Fig. 6 where the current responses of the DIB are recorded for three different oscillation amplitudes (±62.5 μm; ±75 μm; ±87.5 μm), while a frequency of 0.2 Hz is maintained. At each oscillation amplitude, the transmembrane potential is varied between 20 mV and 100 mV. Figure 6 shows the polar plots of different cycles for the three different amplitudes each at a specific transmembrane electrical potential. In the ±62.5 μm displacement case, no gating occurs, where the results resemble those of the channel-free case. This means that the induced bilayer tension is not strong enough to make the channels open. As the amplitude of oscillations is increased to ±75 μm, MscL gating events are observed at transmembrane potentials higher than 80 mV. Similar results are obtained for the ±87.5 μm, however, it is noticed that the conductance levels are higher compared to the lower amplitude case. The results imply that widening of the conductive pore can be achieved through an increase in bilayer tension produced by increased oscillation amplitude. The results presented in this section confirm that both the transmembrane applied potential and the degree of droplet deformation are contributing in increasing the tension in the lipid bilayer membrane.

### Estimation of tension in the interface bilayer

Visualisation of droplets over many compression/separation cycles at different frequencies suggested that flattening and elongation of droplets, as well as changes of the contact angle may generate tension sufficient for MscL gating. We therefore analysed the geometry of the DIB system using image processing techniques for images taken at different positions during the compression cycle. We found that the bilayer contact angle, θ_b,_ decreases while the bilayer contact area increases. At the bilayer interface, to maintain mechanical equilibrium the downward tension (i.e. the bilayer tension γ_b_) should be equilibrated by an upward tension equivalent to the projections of both monolayers tensions (γ_m_) on the plane of the bilayer interface. This relationship is illustrated through the following Young-Dupré equation^22^:


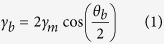


**Figure 7:**
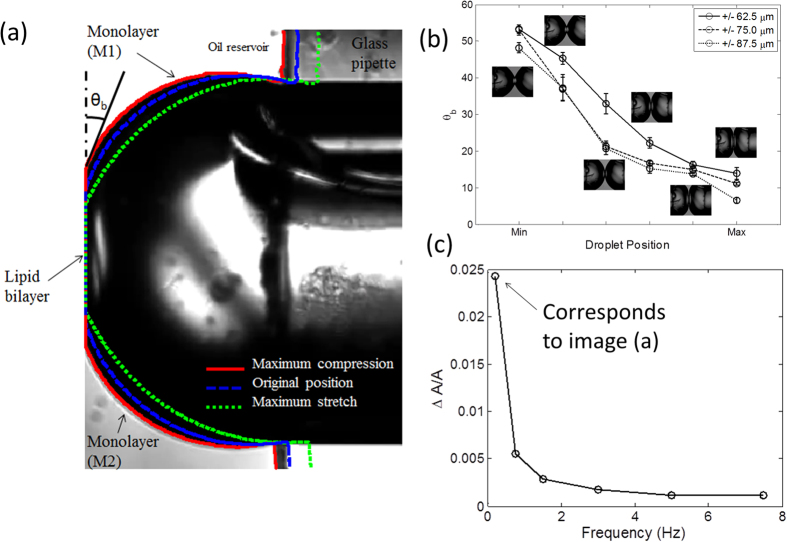
Changes in droplet areas and contact angles revealed by imaging and area calculations in the course of cyclic mechanical stimulation. (**a**) The zoomed-in view of the one droplet and determination of the entire interfacial area, the bilayer area, and the contact angle. (**b**) The bilayer angle as a function of the displacement. (**c**) The relative area changes as a function of frequency of oscillation.

The equation above suggests that γ_b_ is always lower than 2γ_m_, and for this to be true, the Plateau-Gibbs border must move away from the centre of the bilayer in order to increase γ_b_. Note that at the Plateau-Gibbs border, the monolayers (water-oil interface) are near spherical according to the Young-Laplace equation^22^. Therefore, under conditions of constant volume, the area of the monolayer covering the entire water-oil interface in both compressed droplets increases. The relative expansion of the droplets is measured at different frequencies while maintaining a 100 mV transmembrane potential. Our results (Fig. 7c) show that at 0.2 Hz the droplet relative area change is approximately 2.5%. However, as we increased the frequency of oscillation to 7.5 Hz this value dropped to approximately 0.1%. The elastic modulus for isothermal area compression or extension is defined in terms of the mechanical properties of the monolayer as follows:


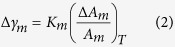


The tension increase in each monolayer can be written as the product of the elastic modulus (*K*_*m*_) and the relative areal expansion of the interface. The compression/expansion elasticity modulus *K*_*m*_ for DPhPC monolayers has been recently estimated as about 120 mN/m^34^. Our results show that with a relative expansion ranging between 2% to 3%, the tension in each monolayer can reach 2.4–3.6 mN/m. These monolayer tension add up and result in 4–7 mN/m in the bilayer membrane which is sufficient to open V23T-MscL^6^. Figure 7b shows the variation in bilayer angle value as the droplets are deformed. In this plot, “Min” refers to the minimum compression point where the droplets are furthest apart, while “Max” refers to the maximum compression point. θ_b_ is measured at different locations between the two extreme droplet positions. The results exhibit a decrease in θ_b_ during compression, which eventually results in an increase in the bilayer tension γ_b_. Another important observation is that the angle at the maximum compression point is decreased as the oscillation amplitude is increased (i.e. the droplets are more compressed). At the point of maximum compression, θ_b_ is small (5–10 degrees), and as a result, γ_b_ is almost equal to 2γ_m_. The bilayer angle being too large, at the point of maximum separation (minimum compression), explains the reason why gating is not seen at the point of maximum separation where the area of the monolayer still increased.

## Discussion

MscL is a well-characterized tension-activated molecular valve. With genetic and chemical modifications of its gates, we can engineer this channel with predicted thresholds for activation by tension. We chose V23T, a mild gain-of-function mutant, which is firmly closed at rest but opens at lower tension than the one needed to gate WT-MscL^6^. We deliberately avoided severe gain-of-function mutants such as V23D or G22E, which tend to gate spontaneously^6^,^35^. We consistently saw V23T-MscL channel activities in response to simultaneous mechanical and electrical stimulation in DIBs. There is a real perspective that such a channel could be used as a transducer in biomolecular stimuli-responsive materials, especially that we found that the protein in reconstituted DPhPC liposomes remained functional for at least three months, and thus it remains stable in liposomes and functional after being reconstituted within the lipid-stabilized oil-water interface.

To date, patch-clamp (which requires bulky equipment) has been the most convenient technique to study the activity of MscL, since it permits clamping of both voltage and tension. However, biomolecular engineering of sensory and conversion devices implies compactness. In DIBs, until present day, tension cannot be clamped and therefore mechanosensitive channels can be stimulated only in a dynamic regime. Despite this, DIBs are emerging flexible platforms that could be adapted to many types of stimuli with the ability to be miniaturized. Here, we present the first study in which the activities of single bacterial MscL channels are directly recorded in a miniature DIB system without the use of a patch pipette or chemical modifications. The developed experimental technique is novel and important since it mimics the natural asymmetry of lipid membranes while changing the membrane tension profile. We successfully reconstituted V23T-MscL channels in the bilayer formed at the interface of two lipid encased droplets and the ability of the DIB to sense mechanical stimuli using bacterial MscL as a mechano-electrical transducer has been demonstrated. The channels self-assembled within the lipid bilayer membrane are activated when tension in the interfacial bilayer is generated. This is achieved by virtue of dynamic droplets’ compression and relaxation through axial sinusoidal displacement of one droplet towards and away from the other. The resulting amplitudes of MscL conductance levels were similar to those recorded by patch-clamp indicating a fully-functional state of MscL in DIBs.

The factors leading to the activation of MscL in the DIB can be categorized as mechanical and electrical. A sufficiently high amplitude oscillation (~20% of the droplet diameter) is required to transmit tension to the bilayer. With a 2.5% maximal observed relative areal expansion of droplets and a monolayer elasticity modulus of 120 mN/m we generated an increase in tension of about 3 mN/m in each leaflet. With small bilayer angles, at peak compression, the increase in monolayer tension roughly doubled in the interfacial bilayer membrane. The estimated peak tension of about 6 mN/m is near the threshold of the V23T-MscL activation curve, which evokes mainly sub-states (Figs 4 and 7) and occasionally fully open-states. Our analysis shows that under the same conditions (i.e. 0.2 Hz frequency, 87.5 μm amplitude, and transmembrane potential of 100 mV) the gating probability per cycle of V23T channels is found to be around 47% (Fig. 7). The distribution of conductance levels across several traces (Fig. 5) shows that the majority of events are sub-conductive states and only in rare occasions (~3%) full openings are achieved. We also found that in the relaxation phase, when the droplets are furthest apart, the area of the droplet interface increased substantially leading to an increase in monolayer tension; however the rise in interfacial bilayer tension is not significant due to large contact angle (~53^o^).

Remarkably, the range of frequencies, in which we were able to see V23T-MscL activities, is narrow. At a frequency of 0.2 Hz, which we found optimal for the chosen geometry, we observed that at peak compression the area of the contact bilayer increased and then decreased by 80% at the point of maximum separation. This “unzipping” of the contributing monolayers allowed the droplets to regain a nearly spherical shape before the next compression phase. The droplets gradually returned to a spherical shape and regained their minimal area (at a given volume), thus enabling higher area and tension increase during the next compression cycle. At higher frequencies, as shown in Fig. 7c, we observe a significantly smaller area expansion due to a delay in the monolayer “unzipping”. Therefore, the viscous resistance of the inter-monolayer gap to flow of the organic solvent (hexadecane) appears to be the natural “low-pass” mechanical filter in the DIB system. However, the presence of the lipid reservoir in the form of liposomes in each aqueous compartment makes the channel only respond to dynamic stimuli, imposing a “high-pass” filter. Transient channel activation, observed under harmonic stimulation, was the way to overcome the membrane tension relief, resulting from the incorporation of the lipid from the aqueous phase into the monolayers. In the course of harmonic deformation of the droplets at 0.2 Hz, the compression phase lasts for 1.25s, during which sufficient expansion of the interfaces and activation tension (~6 mN/m) is achieved. The question is, how quickly and how far the tension may relax due to new lipid incorporation? According to the most recent data, the characteristic time of DPhPC liposome equilibration with the monolayer at the water-oil interface is 180–200s, eventually reaching an equilibrium tension of ~1 mN/m (Dr. Stephen A. Sarles, personal communication). The tension in the compound bilayer at equilibrium is thus estimated to be ~2 mN/m, which is far below the activation threshold for V23T-MscL. However, this relaxation process is slow. Additionally, relatively low lateral diffusion rate of the DPhPC lipid (~18.1 × 10^−8^ cm^2^/s)^36^, will be another factor that slows tension equilibration across each interfacial monolayer. For this reason, tension in the interfacial bilayer will be minimally affected by the incorporation of liposomes from aqueous reservoirs under the chosen 0.2 Hz regime of harmonic stimulation.

Speaking about transmembrane voltage, we found that a 100 mV transmembrane potential is required for the activation of V23T-MscL. Also, the membrane tension may be modulated by the transmembrane voltage^37^. We believe that the combination of different physical parameters, resulting from the mechanical deformation of the droplets and transmembrane voltage, is leading to the gating of MscL in the DIB. It is also important to note that we are dealing with a stochastic and not deterministic behaviour near the threshold, and since we are at the foot of activation curve, we do not expect activation with every cycle.

Further investigation will include understanding the physics of mechanoelectrical transduction in the DIB in order to increase the sensitivity of MscL to a mechanical stimulus. Ways to increase the bandwidth of the system may include miniaturization, reducing the lipid concentration in the bulk to slow down the lipid exchange, chemical cross-linking of lipids, and preparation of DIBs with hydrogels^38^. These findings are fascinating because on one hand, with the use of MscL as a “strain gauge”, we will have the opportunity to understand how tension is created at the interface of a DIB. Additionally, this system will help us learn more about MscL and the ways external forces can be conveyed to its gate.

## Materials and Methods

### Materials

The aqueous phases which form the droplets consist of a suspension of phospholipids vesicles and a buffering agent in highly pure deionized water, while the oil phase consists of Hexadecane (99%, Sigma). The lipid vesicle solution is prepared and stored as described in many articles previously published^27^,^31^. The lipids solution contains 2 mg/ml solution of 1,2-diphytanoyl-sn-glycero-3-phosphocholine (DPHPC, Avanti Polar Lipids, Inc.) vesicles in 500 mM potassium chloride (KCl, Sigma), 10 mM 3-(N-morpholino)propanesulfonic acid (MOPS, Sigma), pH 7. The hydrogel solution with a concentration of 40% (w/v) PEG-DMA contains 0.5% (w/v) Irgacure 2959, and is mixed with a 500 mM KCl and 10 mM MOPS, pH 7 electrolyte solution.

### MscL isolation and reconstitution

V23T-MscL mutant was first generated and characterized by Anishkin *et al.*^6^. The open reading frame cloned in pB10b vector was extended with two PCR steps to add a 6-His affinity tag on the C-terminus. The comparison of activation thresholds between V23T and WT MscL was done in the PB113 *E. coli* strain^39^ carrying native *mscL* gene. The protein was then expressed in MJF465 cells^4^ and purified on a Ni-NTA column (Qiagen) as described previously by Sukharev *et al.*^7^. The protein eluted with 300–500 mM imidazole in the presence of 1% octylglucoside (Calbiochem) was concentrated on Amicon 30 centrifuge filters (Millipore) and its concentration was assayed. An aliquot was mixed with 5 mg of octylglucoside-solubilized DPhPC lipid at 1:500 (w/w) ratio and dialyzed for 48 hrs against 8 liters of 150 mM KCl, 5 mM Tris-HCl buffer (pH 7.2). The formed proteoliposomes were supplemented with 2 mM NaN_3_ and stored at 4 ^o^C. To check the isolated protein for functionality, liposomes were spun down in an Airfuge (Beckman), subjected to dehydration-rehydration cycle and patch-clamped as previously^7^. In parallel, V23T-MscL activities were recorded in giant E. coli spheroplasts as described by Chiang *et al.*^5^.

### Experimental setup

A test setup has been developed in order to characterize the behaviour of the MS channels in the DIB^31^. It consists of two PEG-DMA hydrogel filled micropipettes, an oil reservoir, and a piezoelectric oscillator centred on top of an inverted microscope (AxioSkop-ZEISS). The borosilicate flat tip micropipettes are of 1 mm outer diameter and 0.5 mm inner diameter. The micropipettes are filled with a UV curable hydrogel surrounding a silver/silver-chloride (Ag/AgCl) wires fed into the micropipettes. The hydrogel is cured using free-radical photopolymerization upon exposure to UV light for 3 minutes at 1 W intensity, 365 nm UV source. The micropipettes are then connected to the headstage and the piezoelectric oscillator respectively, and fed through the oil reservoir in opposite directions. The micropipettes are filled with PEG-DMA hydrogel using a 34 gauge, 67 mm long non-metallic syringe needle (MicroFil^TM^) purchased from World Precision Instruments, Inc. Due to the swelling properties of the hydrogel when hydrated, it is very important that it is cured a couple of millimetres away from the tip of the micropipette. Therefore when filled in the micropipette, the hydrogel is sucked back in order form the curved shape. The aqueous lipid and protein mixture is dispensed at the tip of the micropipette using initially the MicroFil^TM^. The droplets of around 0.6 mm in diameter are then formed at the tip of the pipettes through the dispensing of additional liposomes/protein mixture with the help of a sharpened glass micropipette. The droplets sit perfectly within the micropipettes’ tips supported by the hydrogel which will also provide the electric conductivity. Having the micropipettes horizontally opposing each other is meant to maximize the tension at the centre of the artificial membrane where a bilayer with a typical diameter of 0.175 mm (~0.024 mm^2^) is formed.

### DIB formation and recordings

Lipid bilayer interface formed within the biomolecular unit cell is characterized through two types of electrical measurements. Electrically, the lipid bilayer interface is modelled as a capacitor and a resistor in parallel. Therefore, capacitance measurements are carried out in order to verify the increase in capacitance resulting from the bilayer formation. Axopatch 200B and Digidata 1440A (Molecular Devices) are used to measure the resulting square-wave current produced by an external, 10 mV triangular voltage waveform at 10 Hz (Hewlett Packard 3314A function generator). The second type of electrical recording is a current measurement of the bilayer interface, which is held under voltage-clamp while mechanically oscillating the bilayer containing the MS channels. All electrical recordings are carried out under a lab-made Faraday cage that serves as an electrical shield.

## Additional Information

**How to cite this article**: Najem, J. S. *et al.* Activation of bacterial channel MscL in mechanically stimulated droplet interface bilayers. *Sci. Rep.* **5**, 13726; doi: 10.1038/srep13726 (2015).

## Supplementary Material

Supplementary Information
