# Supplementary material for: Activation of bacterial channel MscL in mechanically stimulated droplet interface bilayers
Source: Sci Rep. 2015 Sep 8;5:13726. doi: 10.1038/srep13726 (PMC4562232; doi:10.1038/srep13726)
Supplement: Supplementary Information [file srep13726-s1.pdf]

Joseph Najem <joseph.s.najem@gmail.com>

Permission to cite unpublished work

Sarles, Stephen Andrew (Andy Sarles) <ssarles@utk.edu> Thu, Jan 22, 2015 at 4:34 PM
To: Joseph Najem <jnajem@vt.edu>

Joseph,

Thank you for your email. Because of delays in our publication schedule, the measured tension data on DPhPC at an oil-water interface still is not published. Therefore, I grant you permission to cite as personal communication our unpublished data for the equilibration time (~180-200s) and tension (~1 mN/m) for DPhPC at a hexadecane/water interface.

Please let me know if I can be of additional help.

Regards,
Andy

Andy Sarles, PhD
Assistant Professor of Mechanical Engineering
The University of Tennessee, Knoxville
Dept. of Mechanical, Aerospace and Biomedical Engineering
303 Dougherty Engineering Building
Knoxville, TN 37996
ph: 865.974.8994
email: ssarles@utk.edu

From: Joseph Najem <jnajem@vt.edu>
Date: Thursday, January 22, 2015 10:10 AM
To: Stephen Sarles <ssarles@utk.edu>
Subject: Permission to cite unpublished work
[Quoted text hidden]
